# Nanoscale protein clustering modulates the input–output response of cellular signalling pathways

**DOI:** 10.1038/s41598-026-66329-z

**Published:** 2026-08-11

**Authors:** Luca Panconi, Juliette Griffié, Dylan M. Owen

**Affiliations:** 1 Department of Biochemistry and Biophysics, Stockholm University, Stockholm, Sweden; 2 Department of Immunology, School of Infection, Inflammation and Immunology, College of Medicine and Health, School of Mathematics, Centre of Membrane Proteins and Receptors (COMPARE), University of Birmingham, Birmingham, UK

**Keywords:** Biochemistry, Biophysics, Cell biology, Computational biology and bioinformatics

## Abstract

A key function of the plasma membrane is regulating signal transduction. Proteins involved in the transduction process play a signal-processing role; mapping an input (e.g. number of engaged receptors) to an output (e.g. level of downstream phosphorylation). In many cases, a digital mapping is desirable, i.e. that a cell activates or responds fully once a set input threshold is surpassed. It is hypothesised that the nanoscale organisation of proteins, such as their clustering, modulates this behaviour by altering the frequency of protein-protein interactions. Here, we develop a generalised simulator for dynamic molecular clustering built on agent-based modelling. We show that the clustering properties (e.g. size of clusters, percentage of monomers) tunes the cellular response. This work paves the way for designing potential therapeutic interventions that alter the nanoscale patterning of molecules on the cell surface to influence cell behaviour.

**Supplementary Information:**

The online version contains supplementary material available at 10.1038/s41598-026-66329-z.

## Introduction

Signal transduction across the plasma membrane is vital for several cell functions. This includes, but is not limited to, the function of hormone receptors, transport across ligand-gated ion channels, relocation of T/B receptors of immune cells, and the regulation of adhesion molecules^1–4^. In all cases, a number of proteins must work in concert to transduce the signal and frequently proteins in the signalling pathway will compete – for example, kinases and phosphatases may exhibit opposing functions^5–7^. One study has shown that the interplay between chemical reactions, diffusion, and spatial confinement can influence phosphorylation dynamics in a competing kinase-phosphatase system, leading to geometry-dependent signalling outcomes^8^.

An under-explored aspect of these functions is to consider the input-output, or signal-processing response, of a pathway^9^. The input is the strength of the signal which initiates transduction. This can be interpreted in several ways, but might, for example, be the number of engaged receptors, or the amount of time that those receptors have been engaged^10,11^. The output is then the downstream response. This may include the phosphorylation level of some downstream master regulator, or even changes in gene expression^12^. Single-molecule localisation microscopy (SMLM) has shown that only TCR-CD3 complexes in dense nanoclusters are phosphorylated and associated with downstream signalling proteins, suggesting that local receptor density regulates thresholded phosphorylation events^13^. In addition, it has been observed that TCR phosphorylation, in response to pMHC, is coupled to CD45 exclusion, supporting a physical kinase-phosphatase threshold that can yield switch-like receptor phosphorylation^14^. In general, any relationship between input and output may be possible, but it will be broadly determined by the many proteins that make up the pathway and how they interact including, for example, positive and negative feedback^15^.

It is now widely regarded that many membrane and membrane-associated proteins do not display random distributions on the cell surface. Instead, they show a rich variety of organisations including dimerization, nanoscale clustering and large, microscale aggregates^16–18^. These behaviours are observed despite almost all proteins remaining mobile on the surface with a range of (usually non-Brownian) diffusive behaviours^19,20^. The non-random distributions are usually attributed to a range of biophysical phenomena associated with the membrane including, but not limited to, direct protein-protein interactions, membrane lipid microdomains, corralling by the cortical actin cytoskeleton and active processes like endo- or exocytosis^21–23^.

The nanoscale clustering of proteins is now a measurable phenomenon owing to the development of electron microscopy and, more-recently, super-resolution fluorescence imaging such as SMLM^24^. Combined with statistical analysis, these methods allow for quantification of cluster properties such as cluster sizes, monomer fractions and cluster density, among others^25–29^. The motivation for such studies is frequently that the nanoscale distribution of proteins will modulate the frequency of their interactions (collisions) and therefore can influence the behaviour of the input-output signal processing^3,12^. Despite this, how a particular nanoscale distribution of a protein influences the behaviour of the signalling pathway(s) it is a member of remains poorly understood. Recently, optogenetic clustering tools have been used to probe how nanoscale protein organisation regulates signalling, for example by showing that higher-order Zap70-LAT assemblies, rather than dimers, trigger T-cell activation^30^, or that the order of oncogenic RTK-fusion assemblies tunes Ras-Erk output^31^. However, these methods still cannot finely control how proteins are sized and arranged in space, and in vitro studies are both time-consuming and expensive. In these cases, a computational approach can overcome these drawbacks. Previous studies for example, have shown that the presence of protein clusters can digitise cellular signalling^6^.

Here, we present a simulation ecosystem for modelling protein aggregation dynamics (PAD), based on agent-based modelling (ABM)^19^. Our simulator can generate specified clustered distributions in which all molecules remain diffusive and dynamic. By incorporating multiple protein species (e.g. a target together with a competing kinase and phosphatase), we can use the simulator to test how nanoscale organisation influences the input-output behaviour of a simple signalling pathway. In this case, we take the input to be the ratio of activator (the kinase) to inhibitor (the phosphatase) and the output to be the fraction of phosphorylated receptors. We show that clustering of the pathway’s constituent components does lead to digitisation of the fraction of simulations (cells) that pass a specified output threshold. We further show how the exact clustering properties (such as cluster size) influence this relationship.

## Methods

### PAD simulation software

The ASMODEUS software package (v. 1.0.0) was written in the R programming language (v. 4.4.2) and employed in the integrated development environment RStudio, (2022.07.1 + 554). ASMODEUS is available for use under GNU General Public License (v. 3.0).

### Simulating target distributions

Target distributions were simulated by generating a fixed number of circular clusters, with specified cluster radius and number of points per cluster, then overlaying outliers subject to a given background ratio. Cluster centre coordinates were selected randomly such that all clusters would be contained within the ROI. Cluster points were uniformly generated around centres at distances of $\:{{r}_{t}X}^{2}$, where $\:X \sim Unif \left({0,1}\right)$ and $\:{r}_{t}$ is the target cluster radius. The range of parameter values used in single-population simulations (unless otherwise specified) is given in Table 1.

**Table 1:** Parameters used to simulate target distributions.

Parameter	Function	Values
$\:{n}_{t}$	Number of clusters	10
$\:{r}_{t}$	Cluster radius	10 nm, 30 nm, 50 nm
$\:{p}_{t}$	Number of points per cluster	5, 15, 30
$\:{b}_{t}$	Background to cluster ratio	0, 0.33, 0.66

### General PAD simulations

First, 100 simulations were run over 100,000 frames and the maximum time taken to achieve convergence was measured at 7,529 frames. As such, the maximum frame number for all future simulations was taken at double this value, rounded to the nearest 1,000, to increase the probability of convergence. Therefore, unless otherwise specified, each simulation was run over 15,000 frames, with each frame representing 10 ms of real time, yielding a total simulated time of 2.5 min. All simulations converged within the allocated time. The domain size was fixed at 2 μm × 2 μm, a standard ROI size for super-resolution microscopy. A diffusion coefficient of $\:D\approx\:$ 0.1 µm^2^/s was selected – this represents the diffusion coefficient of a T cell receptor (0.082 µm^2^/s, rounded to the nearest 10^− 1^), taken as a model molecular species^20^. Note that this framework could be applied to any protein with known diffusivity coefficient and TCR serves only as an exemplar here. This equates to a step size of $\:\varDelta\:x\approx\:63$ nm per 10 ms time frame (see Supplementary Material). The average error is recorded at each frame. We define the convergence time as the first frame in which the variance in the error of all future frames fell below 0.05. When applicable, DBSCAN cluster analysis^32^ was performed to extract cluster descriptors, for comparison with the original target parameters.

### Simulating multiple populations

Simulations can incorporate an arbitrary number of distinct molecular species simulated within the same ROI. Species can be simulated separately, by giving each population a separate target and ignoring species outside their own, or co-clustered with other populations, by giving a subset of the populations the same target and only considering the density of species within that subset. Interactions between populations can be recorded and tracked across consecutive frames. In this work, we consider a three-population model, including activators, inhibitors and receptors (which may be active or inactive), depending on interactions with the former two molecular species. Receptors are activated within 5 nm of activators and inhibited within 5 nm of inhibitors. All receptors are initialised as inactive. By outputting the resulting point pattern of any given simulation into another, any of the above regimes can be concatenated.

## Results

### Agent-based modelling as a method for simulating protein aggregation

**Fig. 1:**
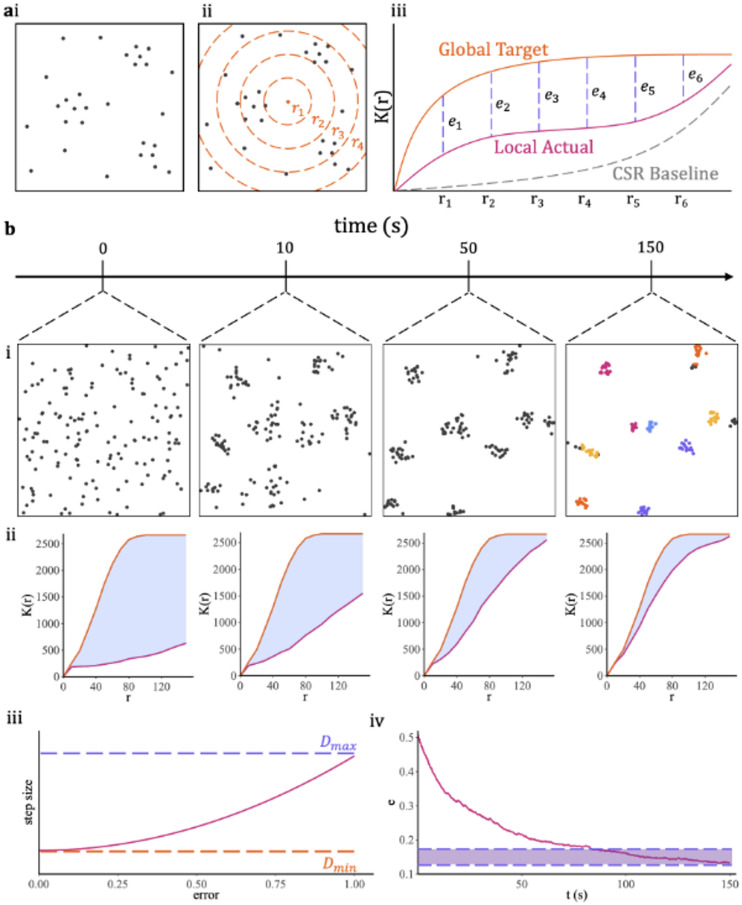
A framework for simulating protein aggregation dynamics. (**a**) An example target point cloud (i) undergoes spatial analysis (ii) to generate a global, target Ripley’s K-function (iii). The error (e) between the cloud’s actual K-function and the target is calculated and averaged at each radius. This error is then mapped to a step size (iv) between minimum and maximum values ($\:{D}_{min}$ and $\:{D}_{max}$) via a quadratic function. Over many iterations, this encourages the K-function to converge to the target, (v) where convergence is defined at the point where the variance of the error falls below 0.05. (**b**) Example of the evolution of a point cloud under this framework (i) and the corresponding Ripley’s K-functions (ii) of the simulation (magenta) and target (orange).

In this work, we build upon ABM approaches and use Ripley’s K-function as a metric to direct the evolution of a point cloud from an initially random distribution towards a target distribution (see Supplementary Fig. 1 for conceptual diagram). Each population is assigned a target distribution generated as a static reference point cloud: this is typically either Complete Spatial Randomness (CSR) or circular clusters of fixed radius overlaid on a CSR background, although it is possible to target any geometric configuration. The localised Ripley’s K function of this reference cloud acts as a target spatial statistic, and simulated molecule coordinates (initialised as CSR) are progressively updated to minimise the error between the dynamic simulated cloud and the static target. The algorithm can be initialised with a (simulated or experimentally-measured) target point pattern or a target K-function (see Supplementary Material). Through an iterative Markov process, each point is sequentially transposed with step size proportional to the error between its own K-function and the global target. The step size is defined by a quadratically increasing function over the error, with minimum step size $\:{D}_{min}$ and maximum step size $\:{D}_{max}$. As the individual error of each point is reduced, so too is the step size, which promotes aggregation at scales summarised by the target K-function (Fig. 1a). This culminates in a model which minimises the error between the measured global K-function of a point pattern and the target. The process is designed to simulate point clouds with defined clustering properties while keeping all points mobile, even after convergence of the measured and target functions (Fig. 1b).

**Fig. 2:**
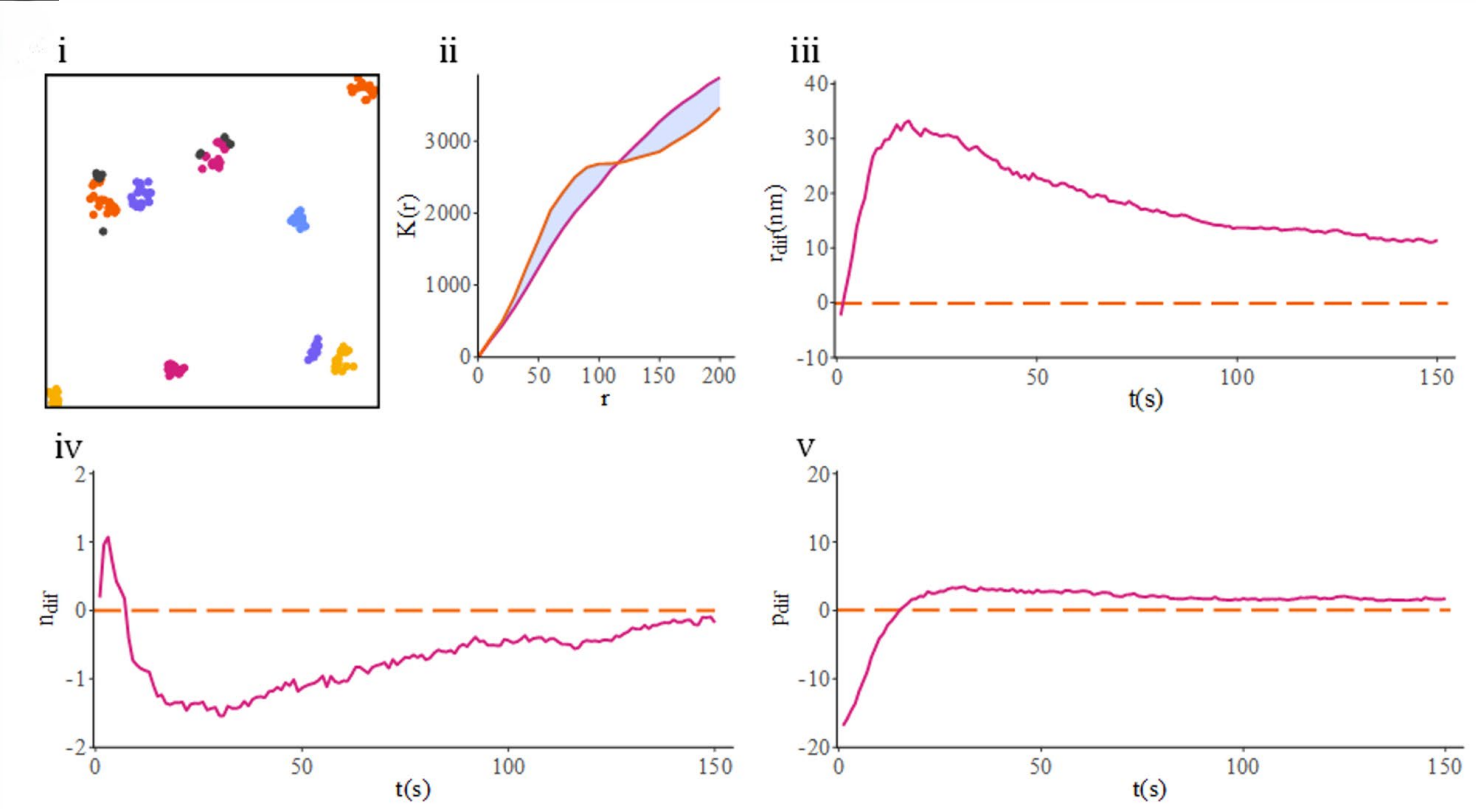
Convergence of cluster properties in simulated and experimental data. Simulated data. (i) Coloured points represent clusters identified by DBSCAN. (ii) The difference between the Ripley’s K-function (orange) and the target function (magenta). Average difference between simulation and target, (iii) cluster radius (r_dif_), (iv) number of clusters and (v) number of points per cluster.

To test the efficacy of our model, we simulated a series of target point clouds with varied cluster properties over 2 μm × 2 μm regions of interest (ROIs). We used a maximum diffusion coefficient of $\:{D}_{max}\:\sim\:0.1\:{\upmu\:}\mathrm{m}{\mathrm{s}}^{-2}$, which equates to a step size of $\:\varDelta\:x\approx\:63$ nm per * 10* ms time frame (see “Methods”). Unless otherwise stated, we fix $\:{D}_{min}=0$. After the model reaches convergence, we then perform DBSCAN cluster analysis to extract cluster descriptors and compare them to the original target parameters^33^. For each simulation, we tracked the point pattern distribution and the measured K-function relative to the target. Further, we record the difference between the measured number of clusters, the measured number of points per cluster, and their corresponding targets over time (Fig. 2). Both the number of clusters and points per cluster converged to within 10% of their input target values.

### Modelling interactions between multiple molecular species

**Fig. 3:**
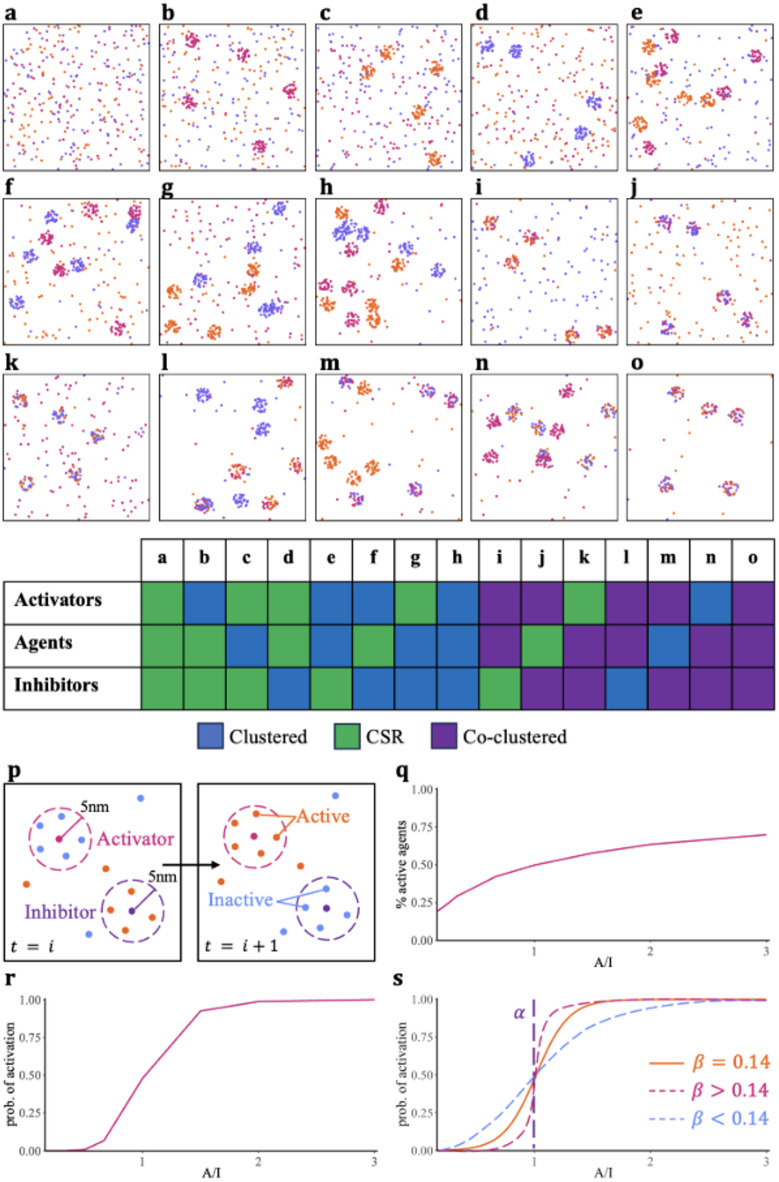
Modelling multiple populations. (**a-o**) Three interacting molecular species under varied geometric point pattern configurations. The geometric distribution of each population is given in the corresponding table. (**p**) Receptors (orange) become active when in a 5 nm radius of an activator (magenta) and remain active until coming within a 5 nm radius of an inhibitor (purple). (**q**) The percentage of active receptors at system convergence versus activator-inhibitor ratios (A/I) for the case for all distributions being CSR. (**r**) Estimated probability of cell activation (percentage of simulations in which the proportion of activated receptors exceeds 50%). (**s**) Fitting a sigmoidal growth function (orange line) to the plot in (**r**) and estimating transition parameters. $\:\alpha\:$: point of inflection, $\:\beta\:$: transition rate (scale). For the fitted line (orange), $\:\alpha\:=1.01$ (purple dashed line) and $\:\beta\:=0.14$. We also show two exemplar sigmoid distributions, given by the magenta and blue dashed lines, which demonstrate the effect of increasing or decreasing the scale parameter, respectively.

Extending upon our single-population model, we have developed a system for simulating multiple interacting molecular species (see “Methods”). Each population may be completely spatially random (CSR), aggregated separately or co-clustered. We consider an arbitrary three-population model, including activators and inhibitors and their receptor targets (which may be active or inactive). We tested each of the 15 geometric configurations given in Fig. 3a-o and described in Table 2. Receptors are activated or inhibited within a specified range (5 nm) of activators or inhibitors, respectively, and remain in their state until acted upon again (Fig. 3p). For each configuration, we considered 64 possible ratios of activator and inhibitor populations and undertook 20 trial simulations for each. During simulation, we varied the number of molecules within each population to adjust A/I ratios. The number of molecules was determined from the simulation parameters (number of points per cluster, number of clusters, and background). For every clustered distribution, a corresponding CSR distribution with the same number of points was generated. As such, the total number of molecules per simulation may vary, but these numbers are consistent across all configurations. This experiment was repeated 9 times, giving a total of 11,520 simulations per configuration, with each simulation lasting 2.5 min. The percentage of receptors which were active at system convergence was recorded (Fig. 3q). The probability of cell activation for each A/I ratio was estimated as the percentage of simulations in which the proportion of activated receptors exceeded a fixed activation threshold of 0.5 (Fig. 3r). A transition function was estimated by fitting a sigmoidal curve (Fig. 3s). From this curve, two parameters were estimated: $\:\alpha\:$, the point of inflection, and $\:\beta\:$, the transition rate or scale (see Supplementary Material for formula). Here, $\:\alpha\:$ may be interpreted as the A/I ratio above which the system is likely to induce cell activation. The lower the value of $\:\beta\:$, the greater the increase in probability of cell activation with respect to the increase in A/I ratio.

Since the percentage of active agents at system convergence generally increases monotonically with A/I ratio, imposing an activation threshold yields an approximately sigmoid distribution, regardless of the choice of threshold. By assuming a constant activation threshold, we can measure the approximate ratio of activators to inhibitors that would result in signal propagation, thereby serving as a mathematical model of signal digitisation, which can be used to approximate the A/I threshold required for signal activation. A further exploration of alternative activation thresholds is given in Supplementary Fig. 2, which highlights the impact on the estimated sigmoid distribution parameters (point of inflection and scale).

**Table 2:** Description of labels for each geometric point pattern configuration given in Fig. 3.

Label	Configuration description
**a**	All populations as CSR.
**b**	Clustered activators, with all other populations as CSR.
**c**	Clustered receptors, with all other populations as CSR.
**d**	Clustered inhibitors, with all other populations as CSR.
**e**	CSR inhibitors, with all other populations clustered.
**f**	CSR receptors, with all other populations clustered.
**g**	CSR activators, with all other populations clustered.
**h**	All populations clustered.
**i**	Co-clustered activators and receptors, with inhibitors as CSR.
**j**	Co-clustered activators and inhibitors, with receptors as CSR.
**k**	Co-clustered receptors and inhibitors, with activators as CSR.
**l**	All populations clustered, with activators and receptors co-clustered.
**m**	All populations clustered, with activators and inhibitors co-clustered.
**n**	All populations clustered, with receptors and inhibitors co-clustered.
**o**	All populations co-clustered.

**Fig. 4:**
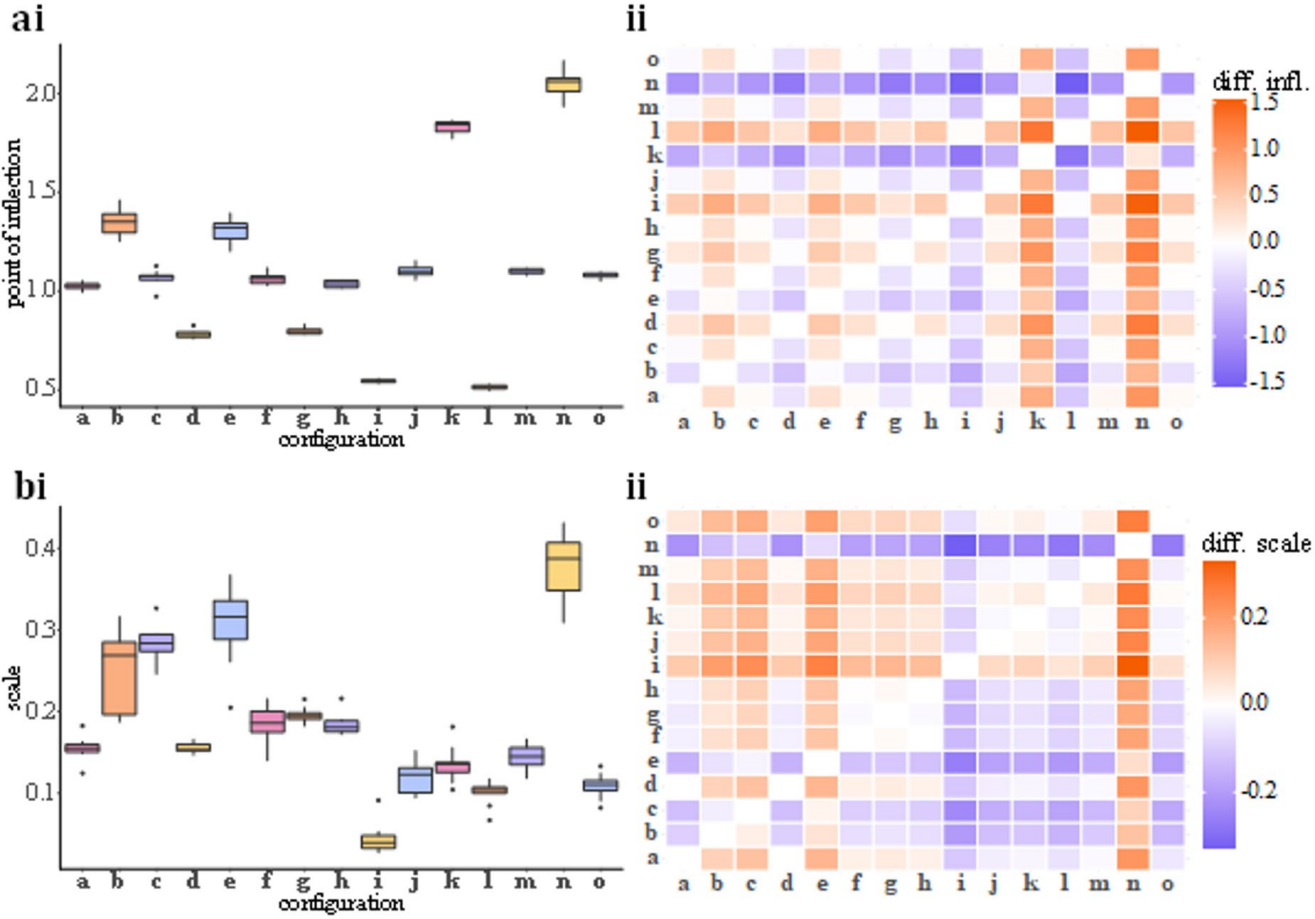
Results of three-population simulations under varied configurations (as shown in Fig. 3). (**a**) (i) Boxplots of all points of inflection estimated from sigmoidal fits. (ii) Differences in point of inflection for each pair of geometric configurations, as returned by Tukey’s HSD test. (**b**) (i) Boxplots of all scale parameters estimated from sigmoidal fits. (ii) Differences in scale parameters for each pair of geometric configurations, as returned by Tukey’s HSD test.

Points of inflection (Fig. 4a) and scales (Fig. 4b) were recorded for each configuration, over all 9 trials. Boxplots depicting the distribution of estimated parameter values are given in Fig. 4a.i. and 4bi. Shapiro-Wilk, Kolmogorov-Smirnov and Anderson-Darling tests were used to assess normality of all estimated parameters across each configuration and for each of the 9 trials. For all cases, there was no statistically significant evidence to suggest non-normality of the data at the 5% level (see Data availability for all p-values). An ANOVA test for evidence of difference between configurations returned a p-value of less than 0.001 for both parameters, suggesting statistically significant evidence of differences in points of inflection and scales between configurations. Tukey’s Honestly Significant Difference (HSD) test was performed for both parameters, across all configurations, to determine individual statistical differences and adjusted p-values between configurations (see Data availability). Results for differences in point of inflection and scale are given in Fig. 4aii and 4bii respectively. We observe the lowest inflection point (i.e. minimum A/I ratio required to induce activation) in configurations i and l, when activators and receptors are co-clustered, and highest inflection point when receptors and inhibitors are co-clustered. Furthermore, we repeated this experiment whilst recording the average step size at convergence (Supplementary Table 1). Here, we find that simulations which have similar average step sizes but different cluster geometries may present highly varied points of inflection and scales. For example, configurations b and d both yield an average final step size of ~ 23 nm/s (relative error between configurations: 0.0004, p value = 0.329 by paired, two-sided t-test), but the corresponding points of inflection are 1.3390 and 0.7784 respectively (relative error: 0.4187, p value < < 0.001 by paired, two-sided t-test). This suggests that observed phase transitions are not simply the result of random diffusion alone, but also depend heavily on the underlying cluster structure. In other words, differences in geometric clustering modulate the inflection (sensitivity) and scale (switch sharpness) of the response.

### Receptor cluster properties regulate signal propagation

**Fig. 5:**
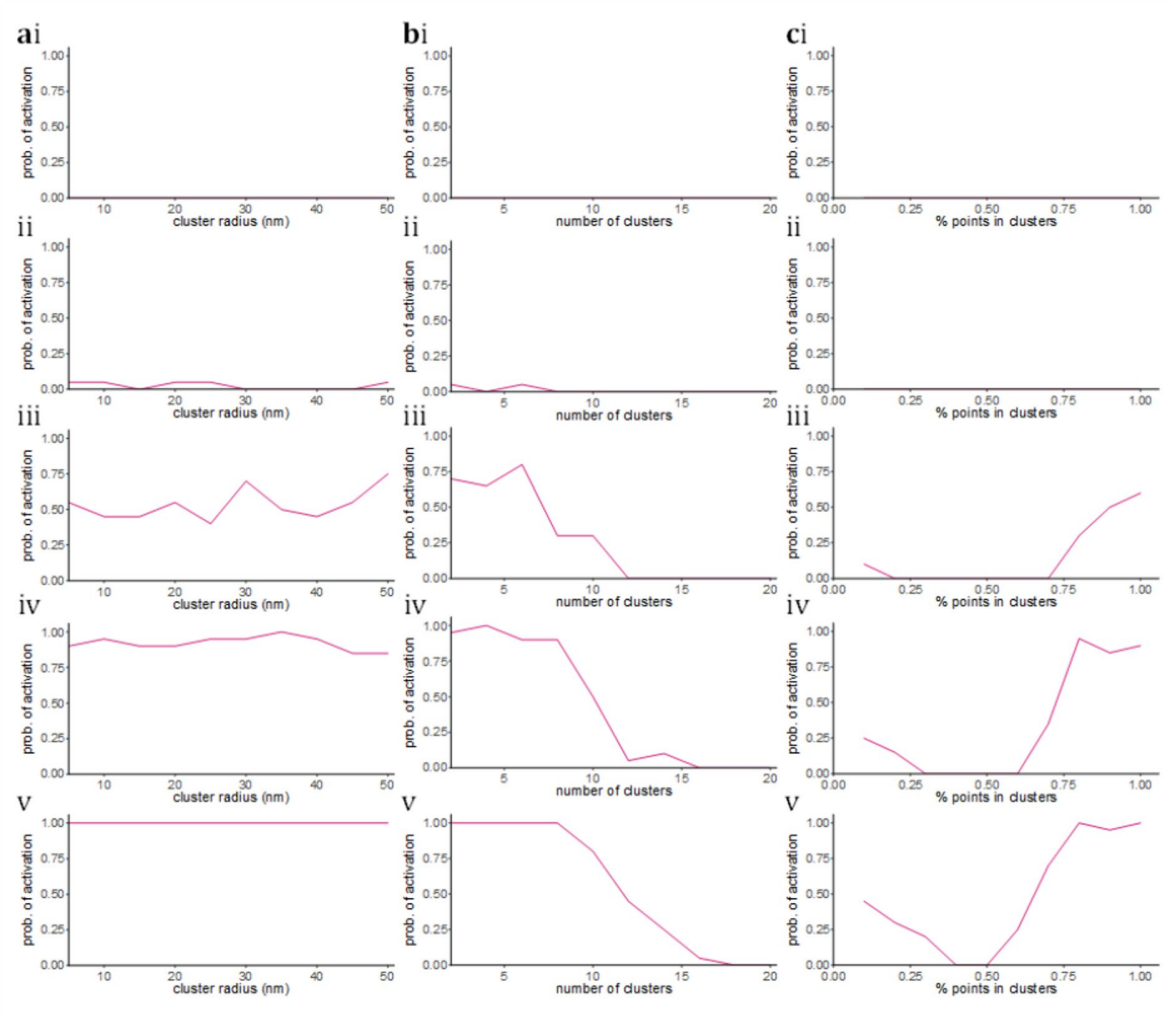
Estimated probability of cell activation versus individual cluster properties in the case where activators were clustered, while all other distributions were CSR. (**a**) Cluster radius versus probability of cell activation. (**b**) Number of clusters versus probability of cell activation. (**c**) % points assigned to clusters versus probability of cell activation. The A/I ratios depicted in this figure are as follows: (i) 0.5, (ii) 1.0, (iii) 1.5, (iv) 2.0, (v) 2.5.

To measure the contributions of each cluster property towards the induction of cell activation, we again consider three-population simulations. Here, we record the estimated probability of cell activation while varying individual cluster properties. For this particular use case, we consider only clustered activators, while all other distributions are taken as CSR. We show the impact of perturbing cluster radius (Fig. 5a), number of clusters (Fig. 5b) and the percentage of points (proteins) assigned to clusters (Fig. 5c) for a range of A/I ratios. For each parameter combination, we generated a static point cloud containing activator clusters (with the corresponding cluster radius, points per cluster and number of clusters). This static point pattern was used as the target distribution for our dynamic, agent-based simulations (where each simulation was initialised with the same number of points as the target distribution). Outputs for the full range of A/I ratios considered in these simulations are given in Supplementary Figs. 3–5.

Results suggest that the probability of cell activation remains approximately constant for cluster radii between 5 nm and 50 nm. Furthermore, increasing the number of clusters generally decreases the probability of activation. This may suggest that activation favours fewer, denser clusters, as opposed to a greater number of sparse clusters. The activation probability also shows a highly non-linear relationship against the percentage of points assigned to clusters. This may suggest that activation probability can be minimised by a trade-off between the percentage of activators within the CSR background and the percentage in clusters. In this case, simulations with a higher fraction of monomers allow the activators to move faster, making them more likely to interact with receptors. This interpretation is validated in Supplementary Fig. 6, in which we record the average step size (per simulation) versus the percentage of points per cluster and show a decrease in step size as the percentage of points in clusters increases.

Furthermore, we repeated this analysis by plotting the percentage of activated agents at system convergence for configurations **l-o**, which can be viewed in Supplementary Fig. 7. We find that increasing overall cluster radius seems to decrease agent activation for configuration **l**, while increasing the probability for configuration **n** (irrespective of A/I ratio). This is expected, as increasing the radius of activator-agent clusters reduces the likelihood of activator-agent interactions, while increasing the radius of agent-inhibitor clusters reduces the likelihood of agent-inhibitor interactions. We observe a similar effect for the number of clusters, as increasing the number of clusters for the molecular species excluded from co-clustering increases the chances of interaction with that species (reducing activation rates for inhibitors and increasing activation rates for activators). This suggests that the analyses herein are robust to the geometric configuration of the molecular species.

## Discussion

Membrane protein aggregation underpins essential cellular processes such as signalling and communication. Central to this notion is the spatial clustering of membrane receptors, which is hypothesised to convert analogue extracellular signals into digital intracellular counterparts. In this work, we have developed an MCMC simulation method to model this process. While the framework is generalisable, here we investigate the simple system of activators (e.g. kinases) and inhibitors (e.g. phosphatases) acting on the same target (e.g. receptors). The simulator allows us to specify a desired cluster configuration for all three molecule types, monitor the receptor activation (phosphorylation) level and then analyse the fraction of simulations (cells) that surpass a specified activation threshold. Many transmembrane proteins form nanoscale clusters, and while it was hypothesised that these clusters convert analogue extracellular signals into digital intracellular signals, the mechanism of digitisation is not fully understood^9^. Here, we have shown that an increase in activator-inhibitor ratio produces a transition function in the form of a sigmoid activation curve, whose parameters vary depending on the cluster configuration being modelled.

Our results show that the clustering properties of the molecules profoundly influence the probability of activation. In particular, the co-clustering of activators and receptors increases the activation probability. Likewise, the co-clustering of inhibitors and receptors decreases the activation probability. The exact level of activation can be fine-tuned by altering the clustering properties (e.g. cluster sizes, percentage monomers and so on). This platform provides a versatile system for testing, evaluating and comparing hypotheses surrounding dynamic reorganisation of transmembrane proteins. In particular, PAD simulations could be used to estimate the geometric properties of phosphatase/kinase distributions which maximise the probability of inducing signal propagation. By modelling receptor distributions under treatment with cross-linking agents or nanotherapeutics (which may be determined from molecular localisation data), it is possible to estimate signalling behaviour without further in vitro experiments. This could be used to inform the design of nano-medicines which explicitly re-organise transmembrane receptors to achieve signal activation (e.g. coated DNA-Origami flat sheets^34^, therefore opening the possibility of developing therapeutic agents which model protein clustering in a controlled way.

## Supplementary Information

Below is the link to the electronic supplementary material.

Supplementary Material 1

## Data Availability

The software package and data that support the findings of this study are available at: [https://github.com/lucapanconi/asmodeus](https:/github.com/lucapanconi/asmodeus) . ASMODEUS v. 1.0.0 and associated data are available for public use under the terms of the GNU General Public License v. 3.0 (DOI: [https://doi.org/10.5281/zenodo.12627125](https:/doi.org/10.5281/zenodo.12627125) ). Results (including p-values) for normality tests and Tukey’s HSD test can be found at [https://github.com/lucapanconi/asmodeus](https:/github.com/lucapanconi/asmodeus) under the directory: /asmodeus/figures/Figure4Panels/.
