# Supplementary material for: Nanoscale protein clustering modulates the input–output response of cellular signalling pathways
Source: Sci Rep. 2026 Aug 11;16:24938. doi: 10.1038/s41598-026-66329-z (PMC13463042; doi:10.1038/s41598-026-66329-z)
Supplement: Supplementary file 1 [file 41598_2026_66329_MOESM1_ESM.docx]

**Supplementary Material**

*Simulating PAD through MCMC agent-based modelling*

Our model is defined under the assumption that proteins are represented by infinitesimal points, which move laterally across the plasma membrane, approximated by a 2D surface. Protein movement is stochastic, with agents moving in a random direction subject to a deterministic step size. This step size, in turn, is controlled by a spatially descriptive statistic (here, the Ripley’s function), derived from the target point pattern. The algorithm for simulating the model is as follows. First, input a target point pattern and appropriate simulation parameters: this includes an expected maximum step size, Dmax, an ROI size, and a time frame over which to run the simulation (for guidance on parameter selection, see below). Calculate the Ripley’s K-function from the target distribution and initialise a completely spatially random (CSR) distribution. For each point, calculate the error between the localised K-function and global target K-function, and then determine the step size (for formulas, see below). Offset each point in a randomly selected direction, apply edge correction to return all points to the domain. A conceptual diagram of the algorithm (**Supplementary Figure 1**) and pseudocode are given below.

*Parameter selection*

Large scale emergent behaviour in ABMs is population sensitive, so the number of agents must be able to reflect the reality of the system being modelled. The precise number of agents found in the target distribution is used automatically. It is generally straightforward to determine the point pattern of protein localisations from raw super-resolution data, from which the molecular counts, ROI size and corresponding spatial statistics are automatically determined in our pipeline. All that remains is to specify appropriate minimum and maximum molecular velocities, proportional to the diffusion coefficient, which can be derived experimentally or mathematically. For applications to experimental data, expected diffusivities can be quantified from imaging methods such as single particle tracking or fluorescence correlation spectroscopy. The expected step size $x$ taken in time $t$ is then estimated by $x=\sqrt{2Dt}$, where $D$ is the diffusion coefficient.

*Limitations of model assumptions and proposed solutions*

The foremost limiting assumptions of our model are as follows:

- Proteins are represented by infinitesimal points and therefore do not collide. This can be circumvented by rejecting protein movements if they come within a range (equal to the expected radius of the molecule) of other proteins and either a) redistributing or b) reflecting the proteins at the angle of incidence, with distance equal to the step size not yet covered.
- The plasma membrane is approximated by a 2D surface and proteins move laterally in the plane of the membrane. However, the Ripley’s function, as a spatially descriptive statistic, is based only on distance and density and can therefore be applied to any number of spatial dimensions. This, with correction for topographic variation, helps circumvent clustering artefacts that could arise from membrane undulations and curvature projected into 2D data^28,71^.

*Calculation of the Ripley’s functions*

The localised Ripley’s K-function for a point $i$ is given by,

$$\begin{aligned} K_{i}\left( r \right)=\frac{A}{n}\sum_{j=1,j\neq i}^{n} \delta_{ij}\left( r \right),\#(1) \end{aligned}$$

where $A$ is the area of the ROI, $n$ is the total number of points, $r$ is the search radius and $\delta_{ij}\left( r \right)$ is the indicator variable that equals 1 if point $j$ is within a radius $r$ of point $i$ (and 0 otherwise). This represents the spatially-averaged density localised around point $i$. Averaging over all points gives the global Ripley’s K-function,

$$\begin{aligned} K\left( r \right)=\frac{1}{n}\sum_{i=1}^{n} K_{i}\left( r \right)=\frac{A}{n^{2}}\sum_{i=1}^{n} \sum_{j=1,j\neq i}^{n} \delta_{ij}\left( r \right),\#(2) \end{aligned}$$

which represents the spatially averaged density across the ROI. The target function derived from the input distribution is denoted $K_{t}\left( r \right)$.

*Derivation of error and step size*

The average ratiometric error of point $i$ is denoted by,

$$\begin{aligned} E_{i}=\frac{1}{\left| R \right|}\sum_{r\in R} e_{i}\left( r \right),\#(3) \end{aligned}$$

where $R$ is the finite set of radial values to iterate over (derived during calculation of the Ripley’s function) and $e_{i}\left( r \right)$ is the scalar error at radius $r$ given by,

$$\begin{aligned} e_{i}\left( r \right)=\left\{ \begin{matrix} \frac{\left| K_{t}\left( r \right)-K_{i}\left( r \right) \right|}{K_{t}\left( r \right)+K_{i}\left( r \right)}, K_{i}\left( r \right)>K_{t}\left( r \right) \\ 1-\frac{K_{i}\left( r \right)-\pi r^{2}}{K_{t}\left( r \right)-\pi r^{2}}, K_{t}\left( r \right)\geq K_{i}\left( r \right)>\pi r^{2} \\ 1, K_{i}\left( r \right)\leq\pi r^{2} \end{matrix} \right\}\#(4) \end{aligned}$$

The lower bound is set at $\pi r^{2}$ as this is the theoretical form of the K-function for a CSR distribution. The set $R$ is granularised for ease of computation and to prevent overfitting. The step size of point $i$ is then given by,

$$\begin{aligned} D_{i}=\left( D_{max}-D_{min} \right)E_{i}^{2}+D_{min},\#(5) \end{aligned}$$

where $D_{min}$ is an optional minimum step size parameter, which defaults to 0. A quadratically decreasing step size was chosen based on previous results^34^.

*Formula for sigmoidal transition function*

The formula for a sigmoidal curve is given by,

$$\begin{aligned} y=\frac{1}{\left( 1+exp\left( \beta\left( \alpha-x \right) \right) \right)},\#(6) \end{aligned}$$

where $\alpha$ is the point of inflection and $\beta$ is the scale parameter.

**ASMODEUS Pseudocode**

#Initialise:

#Dmin - Minimum step size.

#Dmax - Maximum step size.

#ROI - A vector of two values representing the width and height of the region of interest, respectively. The region of interest is considered from the origin, so pointCloud coordinates must be translated accordingly.

#times - Number of time steps or iterations of the simulation.

#pointCloud - A matrix or data frame with two columns: x and y coordinates of each point. Used as a target distribution from which target K function is drawn.

#rmax - Numeric value, the maximum radius for each K function to be calculated at.

#nrval - Numeric value, the number of equally-spaced radial values for each K function to be calculated over.

#target - Manual input for target function. Can be left as null as long as a target pointCloud is provided.

#numberOfPoints - Number of points to use in simulation.

#initialDistribution - The initial frame of the simulation, used to specify pre-determined spatial organisation. If left null, a uniform random distribution will be used.

**INPUT** *Dmin*, *Dmax*, *ROI*, *times*, *pointCloud*, *rmax*, *nrval*, *target*, *numberOfPoints*, *initialDistribution*

#Calculate target K function from pointCloud (if pointCloud is given).

**IF** *pointCloud* is given (not null)

**CALL** RipleyKFunction on *pointCloud*, with r-axis defined on the range of 0 to *rmax* spaced by *nrval* intervals (see **Introduction** for formula)

**SET** output as *target*

**SET** *numberOfPoints* as number of rows in *pointCloud*

**IF** *target* is not given (null)

**END**: not enough information to perform simulation

#Generate initial distribution.

**IF** *initialDistribution* is given (not null)

**SET** *currentCloud* as *initialDistribution*

**SET** *numberOfPoints* as number of rows in *initialDistribution*

**ELSE**

**SET** *currentCloud* as a completely spatially random distribution, defined over *ROI* with *numberOfPoints* points

#Initialise list of frames to store the point pattern at each time frame.

**INITIALISE** empty list *frames*

#Iterate over each time frame.

**FOR** each time *t* in *times*

#Calculate the K function of currentCloud.

**CALL** RipleyKFunction on *currentCloud*, with r-axis defined on the range of 0 to *rmax* spaced by *nrval* intervals (see **Introduction** for formula)

**SET** output as *currentKFunction*

#Calculate error between currentKFunction and target for each point.

#currentErrors is a list containing the average error for each point in currentCloud.

**CALL** errorFunction on *currentKFunction* to calculate error between *currentKFunction* and *target* for each point (see **Appendix** for formula)

**SET** output as *currentErrors*

#Offset position of each point in currentCloud.

**FOR** each point in *currentCloud*

#Calculate offset for each point using stepSize.

**GET** error *e* of point from *currentErrors*

**CALL** stepSize on *e* with parameters *Dmin* and *Dmax* (see **Appendix** for formula)

**SET** output as *step*

**SET** *randomAngle* to be a random number between 0 and 2 * pi

**SET** coordinates of the point in *currentCloud* by moving point along a path of length *step* in the direction of *randomAngle*

#Once all points have been updated, append this new cloud to the list of frames.

**APPEND** *currentCloud* to *frames*

#At the end of this process, frames contains a point cloud for each time frame.

**RETURN** *frames*

| **Label** | **Avg. Final Step Size (nm/s)** | **Avg. Point of Inflection** | **Avg. Scale** |
| --- | --- | --- | --- |
| **a** | 20.77767078 | 1.035319608 | 0.154365134 |
| **b** | 23.11230808 | 1.339023663 | 0.251841803 |
| **c** | 23.06491461 | 1.061818874 | 0.29246226 |
| **d** | 23.02485062 | 0.778356805 | 0.156169379 |
| **e** | 25.34097854 | 1.333881525 | 0.311933935 |
| **f** | 25.39307309 | 1.044646858 | 0.174623759 |
| **g** | 25.2774779 | 0.801772654 | 0.197492262 |
| **h** | 27.58609152 | 1.038334271 | 0.186658978 |
| **i** | 9.857013169 | 0.550126866 | 0.045238172 |
| **j** | 9.853646958 | 1.106587794 | 0.123041922 |
| **k** | 9.859558665 | 1.811961626 | 0.135535054 |
| **l** | 12.11920637 | 0.517710035 | 0.110324012 |
| **m** | 12.13415932 | 1.102988012 | 0.133044075 |
| **n** | 12.19478258 | 2.004299692 | 0.367002303 |
| **o** | 2.840157005 | 1.073726456 | 0.112838157 |

**Supplementary Table 1:** Average step size of simulations described in Figure 3, compared with the estimated parameters of the fitted sigmoid distributions. Configurations of varying cluster geometries may present similar step sizes, but vastly different sigmoid parameters.

Supplementary Figure 1: Conceptual diagram of ASMODEUS algorithm.

 **Supplementary Figure 2:** Estimated sigmoid distribution parameters (point of inflection and scale) for all configurations **a-o**, as described in **Table 1**, versus activations thresholds at values of (0.1, 0.3, 0.5, 0.7, 0.9).

**Supplementary Figure 3:** Estimated probability of cell activation versus cluster radius (with all other parameters fixed) for A/I ratios of **a** 0.25, **b** 0.5, **c** 0.75, **d** 1.0, **e** 1.25, **f** 1.5, **g** 1.75, **h** 2.0, **i** 2.25, **j** 2.5, **k** 2.75, **l** 3.0, **m** 3.25, **n** 3.5, **o** 3.75, **p** 4.0, **q** 4.25 and **r** 4.5.

**Supplementary Figure 4**: Estimated probability of cell activation versus number of clusters (with all other parameters fixed) for A/I ratios of **a** 0.25, **b** 0.5, **c** 0.75, **d** 1.0, **e** 1.25, **f** 1.5, **g** 1.75, **h** 2.0, **i** 2.25, **j** 2.5, **k** 2.75, **l** 3.0, **m** 3.25, **n** 3.5, **o** 3.75, **p** 4.0, **q** 4.25 and **r** 4.5.

**Supplementary Figure 5:** Estimated probability of cell activation versus % points in clusters (with all other parameters fixed) for A/I ratios of **a** 0.25, **b** 0.5, **c** 0.75, **d** 1.0, **e** 1.25, **f** 1.5, **g** 1.75, **h** 2.0, **i** 2.25, **j** 2.5, **k** 2.75, **l** 3.0, **m** 3.25, **n** 3.5, **o** 3.75, **p** 4.0, **q** 4.25 and **r** 4.5.

**Supplementary Figure 6:** Average step size across all simulations versus % points in clusters (with all other parameters fixed) for A/I ratios of **a** 0.25, **b** 0.5, **c** 0.75, **d** 1.0, **e** 1.25, **f** 1.5, **g** 1.75, **h** 2.0, **i** 2.25, **j** 2.5, **k** 2.75, **l** 3.0, **m** 3.25, **n** 3.5, **o** 3.75, **p** 4.0, **q** 4.25 and **r** 4.5.

 **Supplementary Figure 7:** Percentage of activated agents at system convergence versus individual cluster properties for configurations **l-o**, as given in **Table 1**. (i) Cluster radius versus probability of cell activation. (ii) Number of clusters versus probability of cell activation. (iii) % points assigned to clusters versus probability of cell activation. The corresponding A/I ratios are given in the legend.
